# Abnormalities in Skeletal Muscle Myogenesis, Growth, and Regeneration in Myotonic Dystrophy

**DOI:** 10.3389/fneur.2018.00368

**Published:** 2018-05-28

**Authors:** Laurène M. André, C. Rosanne M. Ausems, Derick G. Wansink, Bé Wieringa

**Affiliations:** ^1^Department of Cell Biology, Radboud Institute for Molecular Life Sciences, Radboud University Medical Center, Nijmegen, Netherlands; ^2^Department of Genetics, Donders Institute for Brain, Cognition and Behavior, Radboud University Medical Center, Nijmegen, Netherlands

**Keywords:** myotonic dystrophy, myogenesis, mesoangioblast, myoblast, muscle stem cell, pericyte, proteotoxicity, RNA toxicity

## Abstract

Myotonic dystrophy type 1 (DM1) and 2 (DM2) are autosomal dominant degenerative neuromuscular disorders characterized by progressive skeletal muscle weakness, atrophy, and myotonia with progeroid features. Although both DM1 and DM2 are characterized by skeletal muscle dysfunction and also share other clinical features, the diseases differ in the muscle groups that are affected. In DM1, distal muscles are mainly affected, whereas in DM2 problems are mostly found in proximal muscles. In addition, manifestation in DM1 is generally more severe, with possible congenital or childhood-onset of disease and prominent CNS involvement. DM1 and DM2 are caused by expansion of (CTG•CAG)n and (CCTG•CAGG)n repeats in the 3′ non-coding region of *DMPK* and in intron 1 of *CNBP*, respectively, and in overlapping antisense genes. This critical review will focus on the pleiotropic problems that occur during development, growth, regeneration, and aging of skeletal muscle in patients who inherited these expansions. The current best-accepted idea is that most muscle symptoms can be explained by pathomechanistic effects of repeat expansion on RNA-mediated pathways. However, aberrations in DNA replication and transcription of the DM loci or in protein translation and proteome homeostasis could also affect the control of proliferation and differentiation of muscle progenitor cells or the maintenance and physiological integrity of muscle fibers during a patient’s lifetime. Here, we will discuss these molecular and cellular processes and summarize current knowledge about the role of embryonic and adult muscle-resident stem cells in growth, homeostasis, regeneration, and premature aging of healthy and diseased muscle tissue. Of particular interest is that also progenitor cells from extramuscular sources, such as pericytes and mesoangioblasts, can participate in myogenic differentiation. We will examine the potential of all these types of cells in the application of regenerative medicine for muscular dystrophies and evaluate new possibilities for their use in future therapy of DM.

## Introduction

Skeletal muscle formation, growth, and maintenance in vertebrates are dynamic processes in terms of tissue differentiation, remodeling, repair, and regeneration. During the different phases of life, muscle may suffer due to injury or disease, causing weakness, pain, or paralysis, which may be even fatal. Muscle problems may be acute or short-lived, like during an infection, or be long-lasting, as in chronic disorders. Patients with inherited myopathy or muscular dystrophy, a heterogeneous group of disorders for which disease etiology is rooted in the genetically abnormal pathways that control formation and physiological integrity of skeletal muscle, commonly experience progressive muscle weakness and atrophy (i.e., loss of muscle mass). As a result, physical strength and independence are lost, which causes substantial morbidity over decades. For the development of novel therapies to halt or reverse progression of muscle problems, validated classification criteria for differential clinical diagnosis and detailed preclinical knowledge about what is going wrong at the molecular and genetic level are a prerequisite. Unfortunately, the current states of clinical and fundamental understanding—and hence the prospects for treatment—vary enormously between individual myopathies and dystrophies.

This review is meant to bring new background knowledge for myotonic dystrophy (DM). DM is one of the most prevalent and probably also one of the most difficult to understand genetic disorders, due to its heterogeneity and its highly complex and variable clinical manifestation and molecular etiology. DM is the collective name for a disease with two genetic subtypes, DM1 (OMIM #160900) and DM2 (OMIM #602668). In fact, the classification as a skeletal muscle dystrophy is only partially correct, as the disease also has neuromuscular character and cardiac, CNS and endocrine problems are commonly involved as well (1–3). Here, we will only briefly recapitulate the history of clinical and molecular research in DM as multiple comprehensive reviews have been published on this subject (1, 2, 4, 5). The focus here is on a (re)examination of studies related to the molecular and histomorphological problems that occur during growth, maintenance, and aging of skeletal muscles in patients with DM. Findings in animal model studies are included only if they faithfully reflect the muscular pathophysiology in DM patients (6–8).

The main waves of myogenesis occur during embryonic development and growth, when myoblasts undergo cell cycle arrest and fuse to form the multinucleated myotubes that ultimately become the mature myofibers (9–11). Later, regenerative myogenesis serves in muscle turnover and to replace damaged or diseased muscle (10). Relevance of embryonic and adult stem cells for each of the distinct phases of myogenesis for the manifestation of DM will be examined. We will also describe so-called non-somite skeletal myogenesis through involvement of mesoangioblasts (MABs) and pericytes (PCs) as muscle progenitor cells, and speculate about the importance of this process for DM. Finally, we will discuss possibilities to use these progenitor cells in future therapeutic strategies.

## Myotonic Dystrophy

### Clinical Features and Genetic Causes

A number of clinical and molecular characteristics are shared between DM1 and DM2, but the differences prevail and render them distinct disorders.

#### Myotonic Dystrophy Type 1

Myotonic dystrophy type 1, or Steinert’s disease, shows the highest prevalence, ranging between 0.5 and 18 cases per 100,000 individuals among different ethnic populations (12–14). Progressive muscle weakness and atrophy of the distal muscles together with myotonia are consistent features. Multiple other organs in the body can also be affected, causing combinations of symptoms. For example, heart failure due to conduction problems, insulin resistance, excessive sleepiness, intellectual disability or mental problems, and cognitive deficits are common symptoms (15–18). Anticipation is typical for DM1, which means that disease problems become more severe and occur earlier in successive generations in families. Nowadays, five partially overlapping clinical subtypes of DM1 are recognized, based on the occurrence and onset of the main symptoms: congenital (cDM), infantile, juvenile, adult, and late-onset/asymptomatic DM1 (19). This classification is not only important for patient care but also for the design of clinical trials (2). For a fair interpretation of the literature cited in this review, it is important to note that in studies that appeared before the recent redefinition and refinement of disease classes, authors mostly only discriminated between cDM and adult-onset DM1.

**Figure 1:**
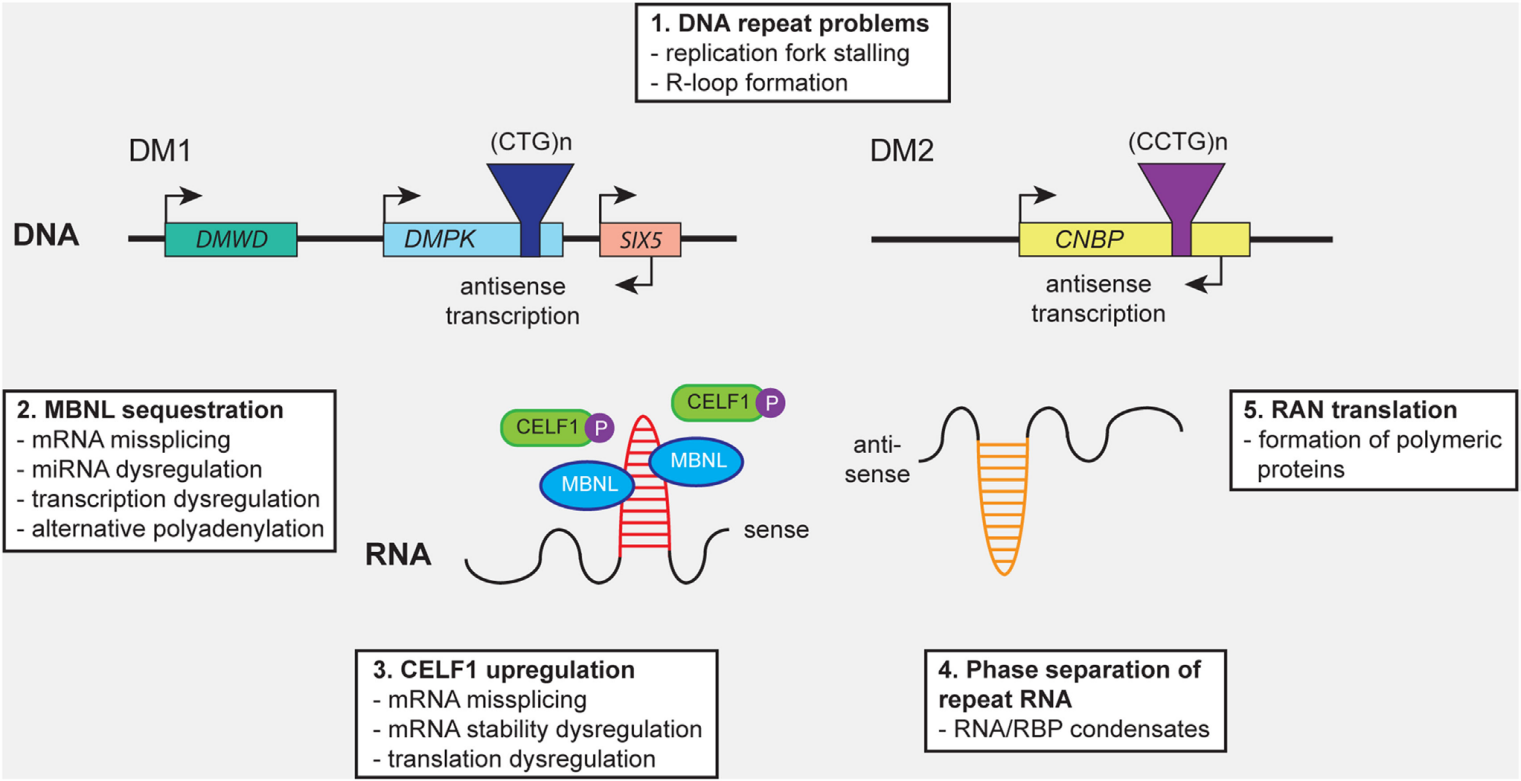
Distinct molecular mechanisms contribute to pathology in myotonic dystrophy type 1 (DM1) and myotonic dystrophy type 2 (DM2). (1) Expanded (CTG)n and (CCTG)n repeats in *DMPK* and *CNBP*, or the complementary repeats in the antisense genes (not shown), can cause cellular stress by (1) promoting DNA replication fork stalling and R-loop formation. Expression of repeat-containing sense and antisense RNAs results in (2) sequestration of members of the MBNL protein family, leading to mRNA missplicing, alternative polyadenylation, microRNA (miRNA) deregulation, and transcription deregulation. In addition, (3) CELF1 gets hyperphosphorylated and stabilized, resulting in mRNA missplicing and dysregulation of mRNA stability and translation. (4) Formation of abnormal RNA-protein condensates by repeat RNA and RNA-binding proteins (RBPs) may alter the intracellular distribution fate and biological activity of RBPs. (5) Repeat-associated non-ATG (RAN) translation of the repeats may result in the production of toxic polymeric polypeptides, which perturb cellular proteostasis.

The sole known molecular cause of DM1 is the expansion of a (CTG•CAG)n sequence on chromosome 19q13 in the last exon of *DMPK* (20, 21) (Figure 1). In DM1 families, when expanded to a length above (CTG)37, the repeat is unstable and has a tendency to grow somatically and intergenerationally (22, 23). Thus, repeat expansion forms the basis for the anticipation phenotype, whereby a longer repeat correlates with more severe symptoms and an earlier disease onset. An expanded *DMPK* repeat is mostly an uninterrupted (CTG)n sequence of variable length. However, additional sequence variations such as CCG and CGG triplets in the 3′ end or immediate flanking DNA, or non-CTG replacements within the repeat have been found. These alterations are generally associated with milder disease manifestation and symptomatic variation in families or seem to occur somatically in certain tissues (24–26).

From the normal and mutant *DMPK* alleles multiple alternatively spliced transcripts are produced, all of which contain the (CUG)n repeat sequence in their 3′ untranslated region (UTR) (27). In addition, there is a partial overlap with an antisense-oriented gene, named *DM1-AS*, which encodes variant (CAG)n transcripts with characteristics of long non-coding RNA (lncRNA) (28).

#### Myotonic Dystrophy Type 2

Formerly known as proximal myotonic myopathy and proximal myotonic dystrophy, DM2 was discovered in a group of patients with clinical features that were slightly different from those in DM1 (29, 30). Prevalence for DM2 varies strongly by population, but is less well known than for DM1, since the mild DM2 phenotype often goes undiagnosed (5). As mutations have been predominantly identified in Caucasians in Northern Europe and this population also has the most registered DM2 patients (31, 32), prevalence of DM2 and DM1 may be quite similar in countries in this region (33). Although the myotonic dystrophies share a number of clinical symptoms, there are distinct differences (34, 35) (Table 1). For DM2 no congenital manifestation is known and diagnosis is always late, when patients have reached adult age. Myotonia is less evident and myotonia of grip often has a jerky quality (36). Proximal muscles are most prominently affected in DM2 and weakness and wasting of facial muscles and limbs is generally mild (29, 30, 36).

**Table 1:** Similarities and differences in genetic, clinical, and histopathological features of myotonic dystrophy type 1 (DM1) and myotonic dystrophy type 2 (DM2).

	DM1	DM2	Reference
**Main features**			
Affected gene, chromosome	*DMPK*; 19q13.3	*CNBP*; 3q21	(20, 32)
Repeat expansion	(CTG)n	(CCTG)n	(20, 37)
Anticipation	Always present	Exceptional	(38)
Age of onset	Any age	Adulthood	(19)
Congenital form	Yes	No	(19)
**Muscle symptoms**			
Predominant muscle weakness	Distal	Proximal	(39)
Predominantly affected muscle fibers	Type 1	Type 2	(40–42)
**Histopathological findings**			
Fiber atrophy	Type 1 fibers (not always present)	Subgroup of highly atrophic type 2 fibers (always present)	(30)
Nuclear clump fibers	In end stage only	Scattered at early stage	(43)
Sarcoplasmic masses	Frequent in distal muscles	Extremely rare	(43)
Ring fibers	Frequent	May occur	(43)
Internal nuclei	Massive in distal muscle	Variable, mainly in type 2 fibers	(43)

Similar to DM1, only one underlying cause of disease has been identified for DM2: all patients carry an expansion of a (CCTG)n repeat in intron 1 of *CNBP* (previously known as *ZNF9*) on chromosome 3q21 (29, 30) (Figure 1). The repeat is part of a complex (TG)n(TCTG)n(CCTG)n motif in which the (CCTG)n repeat is often interrupted and consists of up to 26 units in healthy individuals. In patients the (CCTG)n repeat is usually uninterrupted and contains 75–11,000 quadruplets (36). The DM2 repeat is extremely unstable and has a tendency to expand somatically, causing length increase and cell-to-cell heterogeneity during a patient’s life. Interestingly, by contrast to the behavior of the (CTG•CAG)n repeat in DM1, the (CCTG•CAGG)n repeat has the tendency to contract intergenerationally (44). The correlation between repeat length and disease severity is less strong than in DM1 patients and anticipation is less evident (1, 38).

### Molecular Mechanisms Involved in the Etiology of DM1 and DM2

Several molecular mechanisms are thought to contribute to the muscular pathogenesis of DM throughout all phases of development and maintenance (Figure 1). Similarities with other neurological disorders that are caused by microsatellite expansions have already been comprehensively reviewed (4, 8, 18, 45, 46). Here, we aim to accentuate the relationships between the molecular and cellular levels at which problems caused by the repeat expansions may occur. The emphasis is biased toward the pathobiology of DM1, based on a longer history of study, its seemingly bigger variability and complexity of manifestation, and the broader availability of patient materials, and cell and animal models.

#### Problems at the Chromatin Level

The first level at which repeat expansion may contribute to disease is at the chromatin level. The (CTG•CAG)n repeat in DM1 is situated within the 3′ UTR of *DMPK*, within the overlapping antisense *DM1-AS* gene and in the promoter of *SIX5* (formerly known as *DMAHP*). These genes lie in the center of a gene-rich region of chromosome 19, spanning also *DMWD* (47, 48), *RSHL1*, and *SYMPLEKIN*, within a chromatin loop that is flanked by nuclear matrix attachment regions (49). Two binding sites for the transcriptional repressor CTCF with an insulator role in regulation of transcription and chromatin architecture are within this loop, flanking the repeat area. Already soon after the discovery of the repeat, Otten and Tapscott demonstrated that long (CTG•CAG)n repeats are strong nucleosome positioning elements (50). Extreme repeat expansion as in cDM leads to the occlusion of adjacent DNase hypersensitive sites and concomitant changes in local DNA methylation in the surrounding CG-rich region (51–53), rendering the chromatin more heterochromatic and inaccessible. In turn, this process has *cis-*effects on gene activity in the immediate vicinity, including *DMPK, DM1-AS*, and *SIX5* and perhaps other neighboring genes. To our knowledge, no similar studies of epigenetic changes after repeat expansion in *CNBP* (DM2) exist. Clearly, more work is needed to understand the biological effects that DNA methylation, histone modification and other chromatin changes due to repeat expansion in the DM1 locus have on muscle progenitor cells.

#### Problems at the DNA Level: Stalled Replication Forks and R-Loops

Numerous studies have addressed DNA instability of expanded (CTG•CAG)n and (CCTG•CAGG)n repeats. The influence of oxidative damage and mismatch-repair and recombination pathways for DNA repair on repeat instability have already been thoroughly discussed (54–56). Less attention has been focused on the types of cell stress that large repeats may have at the DNA level and their consequences for loss of cell viability.

DNA polymerase stalling and replication fork arrest seem to be frequent events when unusually large repeat sequences in the genome have to be replicated in S-phase (57). Cells have adequate repair systems to resolve problems with DNA replication fork processivity, either directly when proceeding through the cell cycle or later when they arrive at so-called DNA replication checkpoints (58). Different rescue systems exist in which Chk1 and γH2AX phosphorylation and p53 activation are crucial for the on-site response (58). Stalling at sites in eu- and heterochromatin may even require differential composition of the repair machinery that is recruited. For transcribed repeats, as in the DM1 and DM2 loci, there is an additional complication. Here the threat comes from the formation of so-called R-loops (59). R-loops are triple-stranded RNA-DNA structures formed by duplex formation between the template strand and the transcribed RNA, leaving the non-template strand unpaired. R-loop formation may influence DNA methylation and transcriptional activity in its immediate vicinity. Persistent presence of unresolved R-loops or structures wherein stalled DNA forks and R-loops coincide may affect cellular fitness and arrest the cell cycle. The associated stress may even cause cell death.

An elegant study indeed showed that transcription of a (CTG•CAG)n repeat, as in the DM1 locus, may cause convergent repeat instability and apoptosis (60). Against this background, it is tempting to speculate that proliferating cells in which *DMPK* and/or *DM1-AS* are expressed are vulnerable to the danger of formation of stalled replication forks and R-loops. Specifically, this holds for all mesodermal derivatives and embryonic and adult muscle stem cells [muscle-resident stem cells (MuSCs); see below]. An identical pathogenic cascade may be possible in DM2, since *CNBP* is most highly expressed in muscle (61). There is evidence for bidirectional transcription across the locus (62) and unpaired (CC^T^/_U_G)n or (CAGG)n repeats may form abnormal hairpin structures (63).

#### Misregulation of RNA Processing and Translation

By far the most intensely studied aspects of DM’s etiology are the pleiotropic problems caused by the production of repeat-expanded transcripts. Intranuclear residence of repeat transcripts causes *trans* effects, which culminate in abnormal processing of many other RNAs in the cell’s transcriptome (64).

Probably right after transcription, the repeats in RNAs of *DMPK* and *CNBP* (and the corresponding antisense genes) form stable hairpins that alter activities of two antagonistic protein families, the MBNL (Muscleblind) and CELF proteins. MBNL proteins bind anomalously across the repeat hairpin, leading them to become sequestered in nuclear aggregates, which are visualized as so-called foci under the microscope (65–71). Various other RNA-binding proteins (RBPs) such as hnRNP F, H, DDX5, -6, -17, and Staufen, some of which have intrinsically unstructured domains, are engaged in the nuclear aggregates as well (71–74). CELF1, formerly called CUGBP1, binds at the base of the hairpin and becomes hyperphosphorylated.

Altogether, these events result in an imbalance in cellular ribostasis and proteostasis, associated with depletion and a shift in the distribution of MBNL family members and an increase and redistribution of CELF1 protein. The end result is a cell type and cell state dependent whole-transcriptome effect on alternative splicing (75–78), alternative polyadenylation (79, 80), and nucleocytoplasmic transport of other transcripts for which MBNL1-3 or CELF1 play a role in RNA processing. Changes in mRNA half-life may also occur, as CELF1 has been identified as a key regulator of RNA decay or translational silencing in muscle cells (81). In turn, the changes in the transcriptome have widespread *trans*-acting effects on the production and makeup of multiple proteins (82–86). Some cell-stage effects of MBNL1-3, CELF1, and other ribonucleoprotein (RNP) anomalies will be discussed in more detail below, in the context of embryonic or regenerative myogenesis.

Missplicing may have the most obvious links with the myopathy in DM. For instance, abnormal splicing of *ClC1* is sufficient to cause myotonia (87). Missplicing of the muscle-specific genes *BIN1, TNNT3, RYR1, TTN, LDB3*, and *SERCA1* is linked to impaired muscle function (88). Aberrant splicing of the insulin receptor, highly expressed in skeletal muscle, results in reduced responsiveness to insulin, another contributing factor to skeletal muscle dysfunction (89–91). Furthermore, alternative splicing of *CACNA1S*, a calcium channel that controls skeletal muscle excitation-contraction coupling, is markedly repressed in DM1 and DM2 (92). Combined with splicing alterations in the machineries for voltage-induced Ca^2+^ release and for release and uptake of Ca^2+^ in the ER/SR store (*RyR1* and *SERCA1*), this may lead to chronic Ca^2+^ overload, activate ER stress (93), or become a cause of excitotoxicity. These long-term physiological abnormalities may induce premature senescence and contribute to muscle degeneration in DM.

Not all splicing abnormalities are congruent in DM1 and DM2 muscles. For instance, *TNNT3* is more often misspliced in DM2 than in DM1, and *NCAM1* missplicing can be found more in nuclear clump fibers of DM2 patients (1, 94, 95). Furthermore, in muscle tissue of DM2 patients, *NEDD4* was found to be disrupted. *NEDD4* is an E3 ubiquitin ligase for PTEN, an important regulator of the *AKT* signaling pathway for protection against cellular stress. The PTEN protein level is upregulated in DM2 muscle tissue and PTEN accumulations can be found in nuclear clump 2a fibers in DM2 muscle (96).

For DM2, there may be also a direct effect on ribostasis and proteostasis. Repeat expansion in *CNBP* may cause pausing of transcription or retardation of splicing of its pre-mRNA, resulting in a reduction of mature *CNBP* mRNA and the CNBP protein product. Initial studies on this topic yielded conflicting results, as some groups found unaltered levels of *CNBP* RNA and protein levels in cells and tissues from DM2 patients, whereas later studies showed a clear inhibitory effect of an expanded (CCTG)n repeat (97). Studies on heterozygous knockout mice for *CNBP* brought further support for the idea that haploinsufficiency may be involved in myopathy in DM2 (98). The CNBP protein has a role in the regulation of translation through binding to the 5′ UTRs of terminal oligopyrimidine tract mRNAs. For example, the production of RPS17, poly(A)-binding protein 1, and elongation factors eEF1A and eEF2 are controlled by this mechanism (99).

Also other types of problems at the translational level may play a role in the distinct manifestation of DM1 and DM2. Differential involvement of CELF1 may herein be a key issue. CELF1 can act by relieving secondary structures on a subset of target RNAs that exhibit G-rich sequence stretches with a high-degree of secondary structure, thereby promoting their translatability. Furthermore, if (hyper)phosphorylated, CELF1 may form a multisubunit complex with eukaryotic initiation factor eIF2 and other translation initiation factors, promoting the translation of protein products from alternative start codons in mRNAs that bear an IRES motif (100, 101). Importantly, the different effects of CELF1 on the translation of target mRNAs depend on its phosphorylation status and on the overall level of available protein, which is controlled in accordance with the stage of myogenic differentiation. Although there is no consensus about the fate of CELF1 in DM1 and DM2 muscles, evidence points to a situation in which the available level and thus binding of CELF1 to mRNAs is reduced in DM2. By contrast to the situation in DM1, its phosphorylation status appears unaltered in DM2. When taken combined, these studies support the idea that, superimposed on aberrancies in RNA splicing and polyadenylation, aberrancies in protein translation might have distinct roles in eliciting muscle dysfunction in both forms of DM (99, 102).

#### RNP Condensates: Is Phase Separation of Repeat RNA Causing Cell Stress?

Revolutionary work on polymer physical properties of macromolecular assemblies that undergo liquid-to-gel phase transition and concentration into microscale structures have led to the idea that formation of abnormal condensates by repeat transcripts and RBPs may also be involved in repeat RNA toxicity in DM (103–105). Jain and Vale have recently provided evidence that poly-CUG RNA and also poly-CAG RNA, which both can engage in multivalent intra- and intermolecular reactions, can undergo phase separation *in vitro* (106). They also showed that (CUG)n RNA forms small phase-separated gel inclusions in cells.

More basic studies into the thermodynamics of phase transition have revealed that the threshold concentration at which nano-sized biomolecular RNP condensates are formed are determined by various parameters, including the type, stoichiometry and local concentration of available RNA, and protein constituents and their folding or solubility properties. Most of these studies have been focused on phase transition under conditions with high concentrations of RNA and protein. Future research must thus reveal the requirements for RNA-protein condensate assembly and phase transition in patient cells with endogenous levels of expanded RNAs. Most importantly, the question must be answered whether the occurrence of abnormal repeat RNP gel inclusions containing *DMPK, DM1-AS*, or *CNBP* mRNA with abnormal repeat length could by itself be a trigger for stress.

#### Repeat-Associated Non-ATG (RAN) Translation

Since its discovery in 2011, RAN translation has been linked to proteome abnormalities in multiple repeat-expansion disorders (107). RAN translation of expanded triplet or quadruplet repeats can occur in all reading frames, resulting in the production of homopolymeric (DM1) or poly-tetrapeptide (DM2) proteins (62, 108, 109). In DM1, polyglutamine nuclear aggregates have been identified in myoblasts, skeletal muscle and peripheral blood leukocytes of patients, and in DM1 mouse tissue (108). In DM2, RAN translation across the (CCUG)n and antisense (CAGG)n repeats produces toxic poly-LPAC in neurons, astrocytes, and glia cells, while poly-QAGR proteins accumulate in white matter (62). Whether these findings can be extrapolated to DM2 muscle is an open question.

Many other unanswered questions remain about the production and relevance of RAN products in DM. How does an intronic RNA segment that is normally retained in the nucleoplasm and—without repeat gets quickly degraded—become accessible for the ribosome machinery? A similar question can be asked for DM1, since also expanded *DMPK* and *DM1-AS* RNAs are mainly retained within the nucleus, unavailable for assembly of ribosomes and subsequent translation (28). Nuclear translation is a process that has been demonstrated to occur (110, 111), but at this moment we do not know whether this could be involved. Another possibility is that the initiation of RAN translation occurs only after the onset of prometaphase in cycling cells, so when ribosome subunits are accessible because nucleoplasm and cytosol can mix. Indeed, at mitotic entry, cap-independent translation acquires a dominant role in expression regulation (112). Once polymeric proteins have been produced by RAN translation, they may—alike prion proteins—have a seeding effect in triggering abnormal protein aggregation and condensation and cause imbalance in the cellular proteome (62, 107). This may come at a considerable fitness cost for the cell in which it occurs.

### Cellular Mechanisms Involved in the Etiology of DM1 and DM2

#### Quantitative and Qualitative Aspects Do Matter

**Figure 2:**
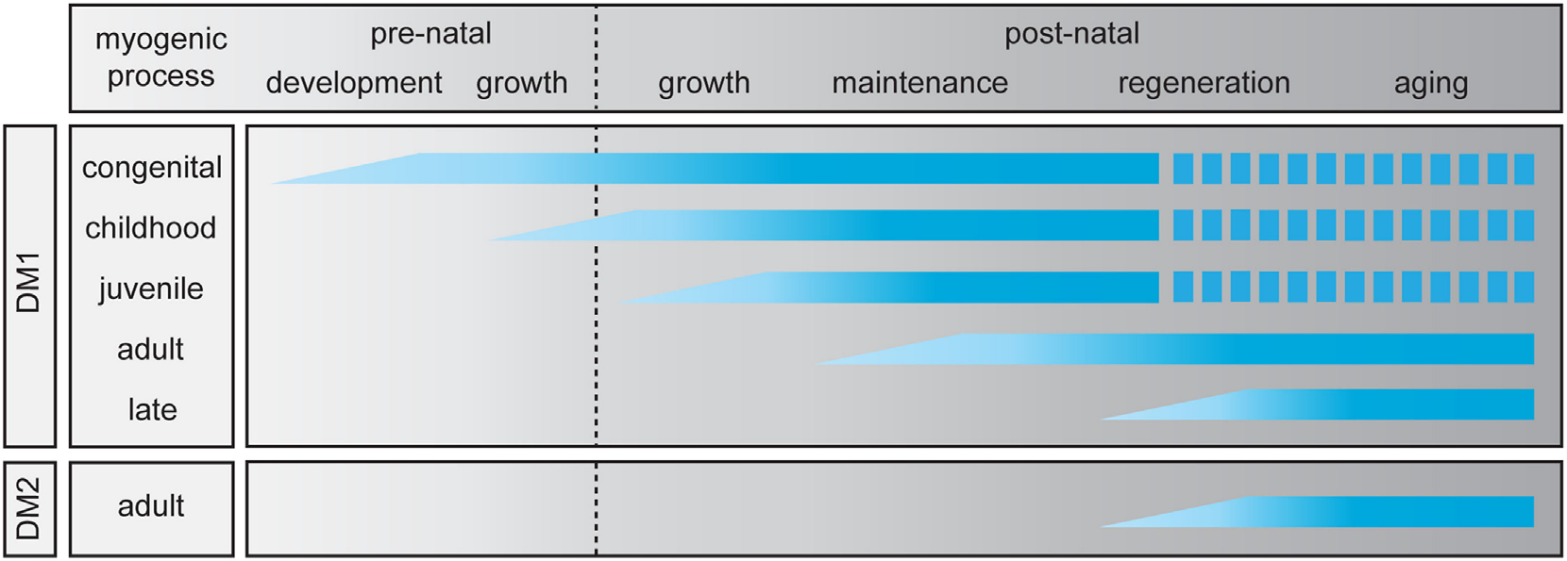
Abnormalities in skeletal muscle myogenesis in myotonic dystrophy (DM). For myotonic dystrophy type 1 (DM1), five clinical subtypes have been identified (19), while for myotonic dystrophy type 2 (DM2) only the adult-onset manifestation is known. The myogenic process in skeletal muscle is divided in two prenatal and four postnatal stages. The graphic summarizes which stages of the pre- and postnatal myogenic process are affected in each clinical DM (sub)type. A discontinuous bar indicates decreased life expectancy.

Any of the molecular disease pathways discussed above could contribute to the myopathy during the different phases of life of patients with DM (Figure 2). However, one should realize that their involvement at the cellular level may differ dramatically with the stage of development and with the type of myofiber that is formed during muscle growth, regeneration, and aging. For example, stalling of replication forks at the (CTG•CAG)n and (CCTG•CAGG)n repeats may not be major threats in quiescent cells, but danger may increase once cells start proliferating. Similarly, reciprocal coupling does exist between the stage and type of differentiation and the mode and extend of alternative splicing in individual muscle progenitor cells or myofibers. The level of *DMPK* and *CNBP* transcripts, splicing factors, or their mRNA targets do, however, vary during muscle differentiation and maturation. So in muscle cells from cDM, DM1, or DM2 patients the complex changes in stoichiometric ratios between MBNL1-3, CELF1, and other RBPs, and the *DMPK* or *CNBP* RNA molecules that take place during natural development are superimposed by variable toxic changes caused by abnormal RBP-repeat RNA interactions (Figure 1).

New supportive evidence for a mutual relationship between differentiation abnormalities and repeat expansion effects was obtained by our group in a study of isogenic CRISPR/Cas9-edited DM1 muscle cells with and without (CTG•CAG)2600 repeat (113). Monitoring of the molecular causes and cellular effect at the individual cell level, during *in vitro* myocyte–myotube differentiation and maturation in culture should thus become possible. Answering the chicken-egg question whether the impaired differentiation and regeneration events or the RNA processing abnormalities and associated cell stress were first in initiating the pathology in DM muscle tissue is not easy. Heterogeneity in cell type composition and developmental stage in the muscle cell population is here the confounding factor. In the next sections, we will try to provide background information on aspects of normal myogenesis and the cellular pathology and histopathology of DM muscle, to come closer to the root of this problem.

#### Muscle Fiber Type and Developmental-Stage Dependent Manifestation of Disease

Within human skeletal muscle there are different categories of fiber types, defined by myosin heavy chain (MyHC) isoform expression and metabolic activity (114). Individual fibers are characterized as one type of slow-twitch fiber (type 1) and three types of fast-twitch fibers [type 2a, 2c, and 2x/d (also referred to as 2b)] (115). Type 1 and 2a fibers are oxidative, whereas type 2c and 2x/d fibers are primarily glycolytic. Type 2 fibers generally produce higher forces and fatigue more quickly than type 1 fibers (116). Walled off from the main part of the muscle in the muscle spindle, highly specialized fibers, known as intrafusal fibers, can be found. These fibers serve as specialized stretch receptors that allow the perception and coordination of limb movement.

Most muscles in the human body are built as a mixture of type 1 and 2 fibers, but between individuals there are marked differences in muscle composition and size. Fiber type content and distribution is thereby coupled to aspects of physical performance, such as endurance and strength. Hence, there is also differential association with disease risk or states between individuals, as skeletal muscle fiber subtypes respond differently to (patho)physiological signals, which include atrophy signals. The ratio of type 1 and 2 fibers within a muscle is altered in muscular disorders when atrophy of one of the two types occurs. Several signaling pathways for muscle atrophy are known, mostly related to abnormalities in protein degradation (117). However, the selectivity of fiber type atrophy remains an unresolved issue (118, 119). For DM, the fiber type specificity of manifestation is a topic that deserves new attention, especially since revolutionary methodologies for transcriptome, proteome, and microscopy analyses at the single cell level have become available.

Skeletal muscles from all DM patients have a distinct histopathological phenotype, but biopsies show conspicuous differences between DM1 and DM2 patients (Table 1). The distal muscles mainly affected in adult DM1 show predominant loss of type 1 fibers (120), whereas the predominantly affected proximal muscles in DM2 show mostly type 2 fiber atrophy (39). Furthermore, an increased variation of fiber diameter and prominent central nuclei with chromatin clumps are present in DM1, normally observed in constantly regenerating muscle with immature fibers (39, 43, 121, 122). Another differential observation is the higher frequency of nuclear clump fibers in DM2. Nuclear clump fibers are typically observed in denervated muscles and have been termed “denervation-like” when observed in DM2 muscle, since other neuropathic alterations were not detected (123). Generally, the alterations seen in muscle of DM2 patients are rather mild and have a heterogeneous character (122). Muscle pathology in DM1 patients has a more typical appearance. However, histological reports, especially of older DM1 studies, may sometimes have a misleading message as researchers usually only draw a distinction between muscles of individuals with cDM and the adult-onset form of disease. Details about graded differences in pathology between muscles from patients with childhood, juvenile, adult, and late-onset/asymptomatic DM1 are not well known.

Already early on it was recognized that cDM is associated with a much broader spectrum of morpho-anatomical muscle problems, with type 1 fiber preponderance and hypotrophy and common occurrence of type 2b fiber deficiency (52, 124). Undifferentiated thin fibers and an increase in satellite cells at birth indicate immature muscle fiber growth and delayed muscle fiber differentiation (125, 126). Also, outside the body in *in vitro* culture the differentiation and maturation capacity of progenitor cells from embryonic muscle of cDM patients was found to be defective (127). The percentage of myoblasts fusing to form myotubes was reduced, the myotube morphology was abnormal, and only immature MyHC protein isoforms were expressed, primarily the embryonic isoform. Also conspicuous aberrancies in intrafusal fiber and muscle spindle presence or morphology were reported. These latter features and the specific fiber type effects may point to additional abnormalities in innervation, motor unit formation, or neurotrophic signaling during the later phases of embryonic development and early prenatal muscle maturation (128–130) (Figure 2).

## How Healthy and DM Muscles are Built and Maintained

### Myogenesis During Early Embryogenesis

The skeletal muscles of limb and torso and head muscles in vertebrates derive from the paraxial and prechordal mesoderm layers in the early embryo. The myogenic process starts when the paraxial mesoderm forms multiple somites, which then further specialize and form the dermomyotome. First, a large proportion of stem cells in the somites and later in the forming limb buds undergo frequent mitosis, under influence of factors such as IGF-1 and PDGF. The proliferating progenitor cells derived from the embryonic mesenchyme of the somite then undergo different phases of myotome development. This process starts with programmed maturation accompanied by adoption of skeletal muscle fate and withdrawal from the cell cycle, giving rise to a layer of non-proliferating myoblasts that form the primary myotome beneath the dermomyotome (131, 132). When more and more cells are progressively added and start to fuse to already committed myoblasts (myocytes) that already reside in the myotome this leads to the formation of the first myofibers and the onset of embryonic muscle growth (133). The following sections will describe the different steps on the road to maturation of skeletal muscles before and after birth.

#### Cell Cycle Exit During Myogenesis

During all stages of contribution to muscle formation and regeneration, myoblasts first need to stop their proliferation process by exiting the cell cycle (134). This occurs by activation of cyclin-dependent kinase (cdk) inhibitor p21 and retinoblastoma protein (Rb), a downstream target of p21. p21 is also partially responsible for the decreased Cdk1 activity observed in differentiating cells (135–137). Formation of Rb–E2F complexes is necessary for maintenance of inhibition of cell cycle progression and for cell cycle withdrawal (138). The role of CELF1 in this regulatory circuit is considered an important link to myogenic problems in DM.

Phosphorylation of CELF1 regulates its intracellular localization and activity. Normally, CELF1 is phosphorylated by AKT and cyclin D3/cdk4 at Ser28 and Ser302, respectively. This posttranslational modification is crucial for myogenic progression. Induction of AKT activity is otherwise involved in the suppression of apoptosis during myogenesis (139). In DM1 myoblasts, CELF1 appears to become hyperphosphorylated by AKT (140), whereas in myotubes, CELF1 phosphorylation by cyclin D3/cdk4 seems to be reduced (141). Alterations in the activity of GSK3β influence the activity in the cyclin D3-CDK4 phosphorylation signaling pathway from upstream. The abnormalities in phosphorylation status compromise CELF1’s role as a translational regulator of a specific population of mRNAs. As an end effect, the changes lead to an increase of cyclin D1, an important regulator of proliferation of myoblasts, and to a reduction of p21 in DM1 myotubes. Together, the changes in the AKT-CELF1-cyclin D1 and cyclin D3/cdk4-CELF1-p21 pathways affect the myogenic process in DM1 (68, 141, 142). Also the Rb–E2F repressor complex appears not to be formed, underscoring that impairment of cell cycle withdrawal may have a role in both forms of DM manifestation (68). However, because not all pathways in which CELF1 is involved are similarly abnormal in DM1 and DM2, other obstructions in myogenic programming might be at play in DM2 as well.

#### Myoblast Fusion

After cell cycle arrest, the fusion of competent myoblasts to form multinucleated myotubes begins. Fusion is a tightly controlled process that involves distinct mechanistic steps, including cell–cell interaction, recognition, and adhesion, followed by membrane coalescence and merging of competent myoblasts to form the multinucleated myotube (143). Extracellular signals from adjacent tissues have an important role in the initiation of several of these steps. Two waves of fusion events take place to form the muscle. Primary myofibers that determine the shape and identity of muscles are formed in the first wave. Secondary myofibers align alongside the primary myofibers and add mass to the muscles in the second wave. Distinct events govern these stages for promotion of differentiation and growth of muscle: first, individual myoblasts fuse to form nascent myotubes and then multinuclear myotubes are formed during subsequent fusion steps between myotubes and additional individual myoblasts (144–146).

The factors that trigger cell fusion (i.e., fusogens) are not precisely known, but numerous proteins that coordinate the formation of primary and secondary myotubes have been identified (144, 146–148). Myomaker, a plasma membrane, Golgi and organellar membrane embedded protein seems crucial (149, 150). Its importance is illustrated by the finding that mutations in myomaker cause a congenital myopathy, Carey-Fineman-Ziter syndrome (151, 152). Other proteins that have an essential role in the myoblast fusion process are myomixer and myomerger. Myomixer, localized to the plasma membrane, associates with myomaker. Myomixer together with myomaker are strong promoters of cell fusion, driving the formation of multinucleated cells from myoblasts (153). Myomerger is only expressed on myocytes and induces the fusogenicity, while myomaker is essential to make a cell fusion competent (148).

Rearrangements in the actin cytoskeleton are first involved in the formation of membrane protrusions between the incoming myoblast and the partner myoblast or myotube. Later they are important for pore formation and cytoarchitectural rearrangements in the resulting multinuclear cell. The entire network that controls the actin network in cells is too complex to discuss here (154, 155), but one issue related to DMPK splice variants may be important. Tentative evidence points to a role for the kinase activity of DMPK, a member of the Rho kinase family, in the regulation of myosin light chain phosphorylation. DMPK may, therefore, functionally link to plasticity of the actomyosin network (156, 157). DMPK is dispensable for myogenesis, as *DMPK* knockout mice are viable and make muscles with only minor abnormalities (156). However, the possibility that *DMPK* splicing becomes spatiotemporally deranged by presence of very long (CUG)n repeats and exerts a modulatory effect on actomyosin cytoskeleton dynamics during early and late myoblast-myotube fusion still exists. Tight regulation of DMPK isoform E during early muscle differentiation is essential for normal development (158) and alternative splicing causes downregulation of DMPK E during myoblast to myotube differentiation (159).

Generally, the muscle problems in adult DM patients are difficult to attribute to any of the distinct phases that determine the differentiation, fusion, or senescence or death of different types of muscle cells *in vivo*. *In vitro* studies on myoblast cultures of adult-onset DM1 with intermediate expansions or DM2 patients are scarce. New methodology was recently published for the immortalization of primary satellite cells, which stimulate *in vitro* studies of differentiation capacity (86). Interestingly, DM2 satellite cells with (CCTG•CAGG)4000 repeats did not have a significantly altered myogenic capacity, confirming earlier findings (66, 160). By contrast, more attention has been concentrated on the study of embryonic or early postnatally derived muscle progenitor cells from cDM muscle. These cells consistently showed impaired myogenic potential and reduced myogenic differentiation capacity during culture *in vitro* (66, 127, 160–164).

#### Transcription Factor-Induced Programming of Myogenic Lineages

To better understand pathological changes in muscle in DM patients, we will first examine the molecular processes that govern normal muscle development (165–168) and discuss these against the background of repeat expansion. The molecular cascade that directs the fate of somite-derived cells during developmental maturation is principally determined by *PAX3* and *PAX7*. These transcription factors trigger the sequential expression of a group of highly conserved myogenic regulatory factors, collectively known as MRFs. MRFs contain a basic helix-loop-helix domain and recognize the E-box in the promoter of target genes (169). *MYF5* and *MYF6* (also known as *MRF4*) act as upstream regulators of *MYOD*, perhaps the best-known member of the family. Co-expression of these three factors is required for myogenic commitment. Then a fourth factor, myogenin (*MYOG*) activates advancement to the myocyte stage and terminal differentiation of the muscle cell (166–168, 170). In this circuit, myogenic transcription factors act in a complex feedback and feedforward network. For instance, the temporal coordination of *MRF*-mediated gene expression is achieved by allowing certain genes to be directly activated by an individual MRF, whereas the induction of other genes in later stages of differentiation by the same MRF requires the participation of the earlier target gene products (166). There is compelling evidence that the expression of various proteins in this MRF regulatory network, like MYOD and MYOG, is affected by the expansions in DM1 or DM2 (69). The involvement of RBPs is thereby a key event. CELF1, for example, binds and destabilizes *MYOD* mRNA *via* its GRE-motif, and an increase in CELF1 activity thus has an inhibiting effect on the progress of myogenic differentiation (72).

Members of the *SIX* family of homeobox genes (*SIX1, SIX2, SIX4*, and *SIX5*) are among the other upstream regulators of MRFs. In mice, *Six4* and *Six5* repress *Myog*, whereas *Six1* activates it (171). *Six1* and *Six4* were shown to be required for *Pax3* and *MRF* expression during myogenesis (172). Interestingly, *SIX5* is immediately adjacent to *DMPK* and its mRNA level seems decreased in DM1 patients (173). *Six5* knockout mice, however, show essentially no muscle symptoms. Hence, the role and relevance of *Six5* in DM1 muscle pathology is not very well established (174–179).

Once it was realized that the coordinate action of transcriptional regulation and alternative splicing (plus other forms of RNA processing) is of key importance for myogenic development (72, 75, 180), also the role of isoforms of accessory transcription factors in impaired muscle differentiation in DM attracted further attention. First evidence for their significance came from a study of members of the *MEF2* family. In vertebrates, four members of this family, *MEF2A, -B, -C*, and *-D*, are expressed. Although MEF2 members do not possess own myogenic activity, they act together with MRFs to activate and sustain the myogenic differentiation program (85, 181). As discussed earlier, MBNL1, -2, and -3 are key factors in the missplicing in DM. In their normal role, MBNL1 and -2 are positive regulators of muscle differentiation. MBNL3, on the other hand, inhibits muscle formation, by repressing adult mRNA splice isoforms (182–185). Lee et al. showed that MBNL3 influences myogenesis by disrupting *MEF2D* splicing, by favoring beta-exon exclusion (186). When the beta-including MEF2D isoform was expressed in a cell model, normal muscle differentiation was restored. Almost coincidentally, others reported on splicing changes for *MEF2A* and *-C* mRNAs. Dysregulation of *MEF2B* and *-D* and genes that are under transcriptional control of these factors, mainly those involved in calcium signaling, was found as well (88). Likewise, CELF1 upregulates translation of *MEF2A* mRNA *via* direct interaction with a GC-rich element in the transcript, causing a delay in myogenesis. Abnormal CELF1 upregulation thus explains the muscle maturation delay in DM1. For DM2 the involvement of coupled transcription-RNA processing abnormalities has not yet been documented.

#### First Appearance of Committed MuSCs

During early embryogenesis, a subselection of cells from the dermomyotome maintains proliferation and migrates directly to the myotome. These *PAX3*- and *PAX7*-positive cells do not express members of the MRF, homeobox or *MEF* families of transcription factors. These cells are known as the myogenic precursors that form the source of the majority of satellite cells in the adult skeletal muscle, and as such form the subject for further discussion in the next sections.

### Embryonic and Prenatal Phases of Muscle Growth

#### Fiber Type Specification

In most vertebrates, fibers of diverse types are recognized in the embryo concomitantly with the earliest time points of muscle appearance, before innervation (187). Interestingly, slow MyHC-expressing fibers seem to form earlier than fast MyHC-expressing fibers. Hedgehog signaling is a determining mechanism required for muscle precursors to commit to the slow muscle fate. Later in development, beyond the late embryonic and fetal periods of prenatal development, the slow (type 1) fibers become less common and fast fibers (type 2) start to become the most abundant fiber type. External soluble signals, such as WNT, coming from tissues adjacent to the somites—i.e., the notochord and neural tube—plus cell–cell contacts in the embryonic niche play an important role in further growth of muscle and the specification of fiber types. Excellent reviews discuss the regulatory principles behind fiber specification (180, 187, 188).

The functional and architectural properties of fiber types that arise during embryonic and fetal muscle development are with the advancement of growth further modified by effects of physical activity, endocrine signals and muscle innervation (180, 187–189). This process continues further during postnatal life. For a better understanding of the distinct fiber type involvement in DM1 versus DM2, it is important to reiterate here that not only type 1 and 2 fate specification but also intrafusal fiber morphogenesis is under control of new combinations of transcription factors. Transcription factor EGR3, for example, is selectively expressed in sensory axon-contacted myotubes, and is a key factor for normal intrafusal fiber differentiation and spindle development (190–192). *ERB2* signaling also plays an important role (193). As was specified above, intrafusal fiber and spindle morphology are clearly affected in cDM muscles.

Similar hierarchical networks determine the fast and slow fiber specification. Involvement of transcription factors PRDM1 and SOX6 has already been well documented. PRDM1 acts as a switch that activates the slow-twitch differentiation program in cells by direct repression of fast-twitch specific genes and indirect activation of slow-twitch specific genes through limiting the activity of the SOX6 transcriptional repressor (188).

During the transition from the embryonic to the fetal phase of development, a switch occurs from basic muscle patterning (primary myogenesis) to growth and maturation of the muscle masses and the onset of innervation (secondary myogenesis). These two waves of myogenesis are mediated by distinct embryonic and fetal myoblasts, respectively, each characterized by differentially expressed genes and properties. The differentiated cells that these myoblasts produce later have also distinct features. Expression of *NFIX* is an important prerequisite for the continuation of coordination of fiber specification in the switch to fetal muscle growth. Gradual changes in the networks for transcription regulation, alternative splicing and polyadenylation thereby jointly control the differential expression of fiber type specific protein isoforms. Single muscle fiber proteomics studies have revealed hundreds of proteins that vary in level or identity between the proteomes of different fiber types. Among these are protein isoforms involved in sarcomeric architecture, contractile activity, mitochondrial and carbohydrate metabolism, calcium handling, and protein turnover (194). Differential activation of genes for fiber type specific isoforms of myosin, troponin, tropomyosin, creatine kinase, B-enolase and glycolytic, and mitochondrial enzymes is typical in this specialization (195).

Until now, not much attention was paid to differential expression of genes whose products are linked to the RNA toxicity mechanism in DM. To the best of our knowledge no publications exist on differences in expression of *MBNL1-3* or *CELF1* between fast and slow fibers. Also reports on abnormalities in expression of *DMPK* and *CNBP* in individual fiber types in DM1 or DM2 are rare. In one early report, a decrease in *DMPK* expression in type 2a muscle fibers of DM1 patients, compared with the level in normal controls, was mentioned (196). Wheeler et al. have reported an abnormal foci count in subsynaptic nuclei and in nuclei of motor neurons at muscle-nerve junctions (67).

#### Muscle Progenitor Cells of Different Origin

During late fetal development the fiber composition of muscle is further defined and profound changes in the direct neighborhood of the muscle occur. Within the basal lamina formed around the muscle, the fibers are now located together with the now quiescent population of PAX3+/PAX7+ MuSCs, the satellite cells. At the end of the fetal period ~30% of myonuclei are satellite cell nuclei. The remaining ~70% are in the multinucleated fibers. Blood vessels permeate the interstitial spaces between fibers and nerve endings have established contact *via* neuromuscular junctions. During the transition to adulthood, the percentage of mononucleated cells located under the basal lamina at the muscle periphery declines sharply, due to recruitment for muscle growth and maintenance. In adult muscle, the population of satellite cells encompasses 2–5% of identifiable nuclei (11, 197, 198), which declines further during aging.

**Figure 3:**
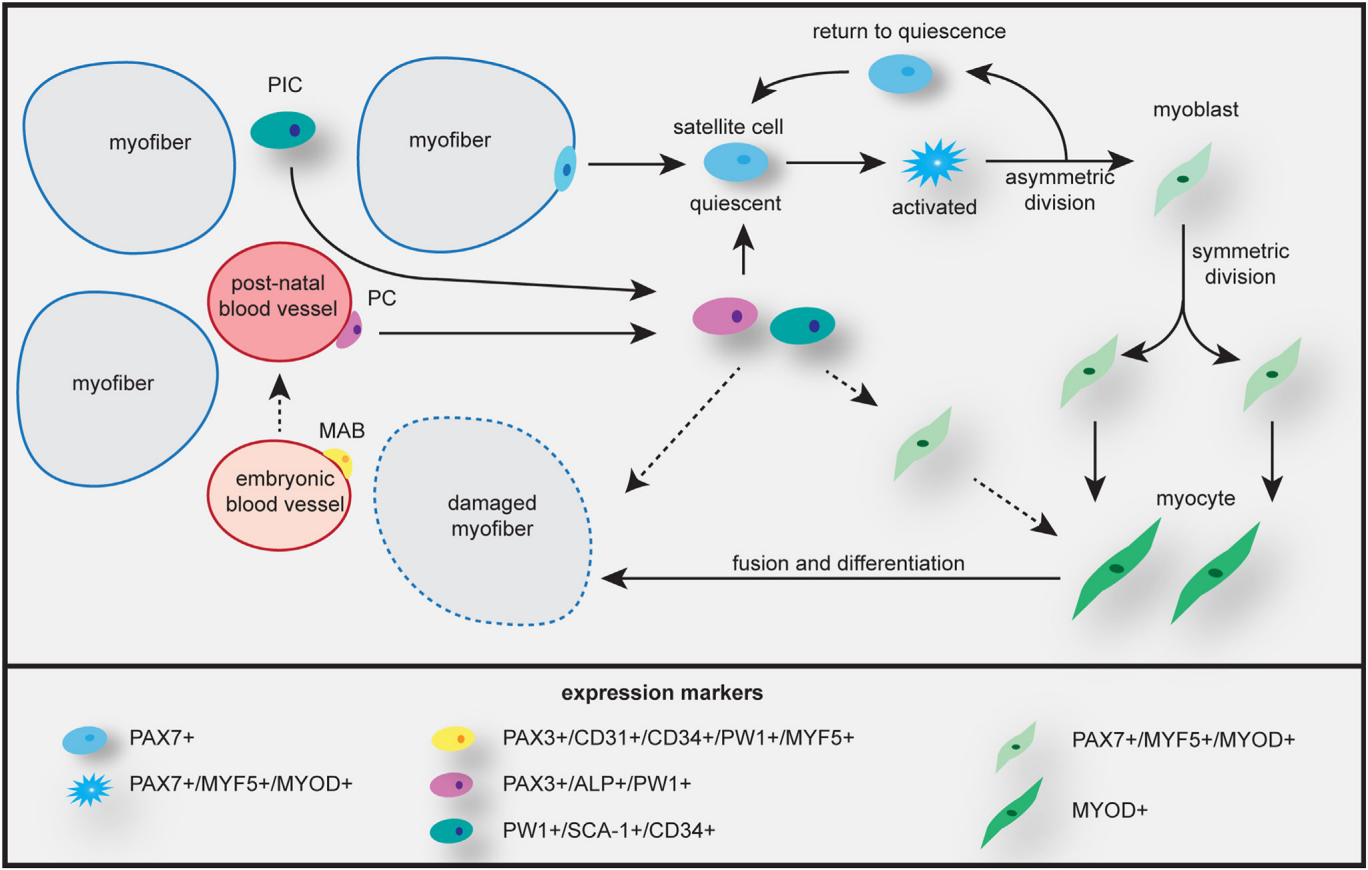
Skeletal muscle growth, maintenance, and repair by different myogenic progenitor cells. Satellite cells from the basal lamina of the myofiber are activated and undergo asymmetric and symmetric division to generate heterogeneous progeny. Some cells undergo self-renewal and return to quiescence, others become myoblast that will proliferate and differentiate to become myocytes, which fuse to myofibers, enabling repair and/or growth. Mesoangioblasts (MABs) can contribute to muscle regeneration during embryonic growth, while pericytes (PCs) are involved in postnatal muscle growth by repopulating the quiescent stem cell population or maybe by transforming into a myoblast. Participation in growth and/or repair or direct fusion with the myofiber probably occurs along the same pathways as given for satellite cells. Uncertainties in cell fate are indicated by dashed arrows. PW1+ interstitial cells (PICs) are mostly involved in perinatal growth. Expression signatures of differentiation markers in all different cell types are listed at the bottom.

There is now compelling evidence that the skeletal muscle niches thus formed contain multiple types of cells, among them also cells with non-somitic origin, with myogenic capacity (Figure 3). Together with the satellite cells, the major skeletal muscle progenitor/stem cell population, these cells form the reservoir for use in skeletal muscle repair, regeneration, and maintenance (Table 2). Specifically, different interstitial populations of cells have now been characterized, referred to as PW1+ interstitial cells (PICs, that express *PW1/PEG3*) and β4-integrin+ cell (199). Other progenitor cells, MABs and PCs, are located in the fetal or postnatal muscle vasculature, respectively. PCs express alkaline phosphatase (*ALP*), but lack myogenic and endothelial markers (200). Using lineage tracing, it has been shown that most of these non-satellite cells are not derived from the somite, as do the true PAX3+/PAX7+ satellite cells. The vessel-derived progenitors can be traced back to Pax3+ progenitors of the paraxial mesoderm (201, 202). Both the muscle-resident satellite cells and the PCs contribute to muscle growth during prenatal and postnatal development.

**Table 2:** Myogenic cell types.

Cell type	Abbreviation	Definition
Muscle-resident stem cell	MuSC	Collective term for cells in (adult) skeletal muscle that can self-renew and give rise to muscle cells
Satellite cell	–	Muscle progenitor cell located in the adult stem cell niche under the basal lamina of the myofiber; upon muscle injury this cell can undergo symmetric or asymmetric cell division and produces cell progeny that undergo self-renewal or become myoblasts
Myoblast	–	General term for a mononuclear muscle progenitor cell that can proliferate or undergo terminal myogenic differentiation
Myocyte	–	Quiescent differentiated myoblast that can fuse to a myotube
Myotube	–	Multinuclear cell formed by the sequential fusion of myoblasts/myocytes, which will develop into a mature myofiber
Myofiber	–	Mature multinuclear muscle cell; the smallest contractile unit of a muscle
Induced pluripotent stem cell	iPSC	Pluripotent stem cell generated from an adult tissue cell (often a fibroblast)
Mesoangioblast	MAB	Cell isolated from the embryonic microvascular wall. A MAB has the potential to self-renew and generate multiple types of differentiated cells
Pericyte	PC	Cell isolated from the microvascular wall of postnatal tissue. A PC is capable of (trans)differentiating into other cell types when naturally or experimentally relocated to a different tissue

We will next examine the role and fate of satellite cells in growth, renewal and regeneration of muscle. The biological significance of the other progenitor cells introduced above will be discussed further below in the context of regenerative medicine. DM pathobiology has only been studied in the satellite cell-derived myoblast population *in vitro* and by histological examinations *in vivo*. No data exist on the involvement of PICs, MABs, and PCs.

### Muscle Renewal and Regenerative Myogenesis

Skeletal muscles endure a lot throughout a lifetime. First, muscle tissue has to grow in size. Then it must be constantly functionally and structurally renewed and maintained in accordance with physical demand and repaired after injury or disease. The role of the satellite cell compartment is thereby indispensable. The mechanisms by which satellite cells participate in renewal and regeneration of muscle have overt similarities to developmental myogenesis. Satellite cells follow largely the same trajectory as somite muscle cells during development, except for their start, which begins in a state of mitotic quiescence. The population of satellite cells must also be kept in check, to maintain functionality and to guarantee muscle homeostasis up to high age. This necessitates maintenance of a delicate balance between self-renewal and differentiation. In fact, evidence has accumulated showing that distinct satellite cell pools in anatomically defined muscles in the body are heterogeneous cell populations, with cells in different stages of development having different gene expression signatures (167, 199, 203–205).

#### Maintaining Tissue Homeostasis in Adult Muscle

In reaction to disease, injury or prolonged hypoxia, the local release of cytokines, growth factors, cell differentiation factors such as NOTCH and WNT, and other signals triggers satellite cells that are in a quiescent state. The muscle tissue itself and nearby fibroblasts and macrophages have a role in this process. The signaling starts a program of re-entry of satellite cells in cell cycle. Subsequent rounds of cell division, combined with differentiation programming, along similar lines as in embryonic development, in a subset of the satellite cells produces heterogeneity in the population. Some satellite cells retain stemness, and others become myoblasts or myocytes that undergo definite differentiation commitment (Figure 3).

Expansion in the muscle stem cell niche assures that some cells can remain associated with the extracellular matrix and to cells in the neighborhood. This promotes polarization and allows different cycles of asymmetric cell division, maintaining undifferentiated satellite cells, ready for reversal to quiescence (requiescence), and committed progeny for differentiation. Cells with highest expression of *NUMB*, an antagonist of *NOTCH* signaling, go back in quiescence for later self-renewal (206). Daughter cells in which p38α/β MAPK is asymmetrically activated by a so-called PAR complex, undergo commitment to myogenic differentiation (207), expand in number and form binuclear myotubes or fuse to existing fibers (168, 199, 203, 208).

A general repression of translation, mediated by the phosphorylation of translation initiation factor eIF2α, is also a key event in the maintenance of the quiescent state (209). The mitotic quiescent satellite cells express *PAX7, MYF5*, and *CD34* and frequently also *PAX*3 (210–214). Entrance in cell cycle and progression through the myogenic lineage occurs under the control of MRFs. Activated satellite cells no longer express *CD34* and start expressing *MYOD*. Once activated satellite cells proliferate and become myoblasts, *PAX7* expression is downregulated, while *MYOD* and *MYF5* expression remain (215). *In silico* modeling of RNA processing associated with human muscle development has provided strong evidence that also the expression of *MBNL1, -2*, and *-3* varies during these transitions in cell state (85). In addition, *MYOD* induces the expression of p21. As mentioned earlier, p21 blocks cell cycle progression and it is involved in the switch from proliferating to differentiating myoblasts, i.e., when they become myocytes. This switch is essential for myogenic precursor cell, satellite cell, function in regenerating skeletal muscle (135, 216). During normal healthy life, this whole cascade of steps for the regulation of muscle differentiation and maintenance is orchestrated by a multitude of circulating hormones, such as IGFs, FGFs, TGFs, testosterone, thyroid hormones, cytokines, and exosome-secreted signals, which are secreted locally and appear in the muscle stem cell niches. Whether and how hormonal signaling controls viability, performance and half-life of multinucleated myofibers—i.e., the bulk of muscle mass in a healthy individual—is still poorly understood, as attention of study thus far has been mainly directed toward MuSCs (208).

#### Failure of Tissue Homeostasis in DM Muscle

Not much is known about the fate specification of terminally differentiated multinucleated myofibers in DM. One likely possibility is that the persistent abnormalities in alternative splicing, alternative polyadenylation, and unscheduled translation of aberrant transcripts lead to the production of excessive amounts of ectopic proteins. When combined with a bulk of proteins synthesized in normal accordance with the stage of muscle during adulthood, this will create a permanent disbalance in the assembly—and perhaps turnover—of multiprotein complexes in the fiber proteome. Production of polymeric proteins by RAN translation may further create proteome abnormality. Ultimately, such imbalance will lead to a culmination of problems and to proteotoxic stress alike UPS, ER stress, or other forms of stress mentioned in this review (217). When certain thresholds are exceeded, this may lead to senescence or apoptosis. Somatic expansion of repeat length during aging may further augment the stress level, causing loss of an increasing number of fibers with disease progression and aging.

Why pathology specifically involves type 1 fibers in distal muscles of adult-onset DM1 patients and type 2 fibers in proximal muscles in DM2 needs more study. The answers may not be found only in the mature fibers themselves. They also must be sought in differences between DM1 and DM2 in the fitness of their satellite cell pools, or in the modes of recruitment of satellite cells for the regeneration of damaged fibers. As addressed before, the relevance of the satellite cell pool becomes early apparent in cDM patients, who are born with an excessive number of satellite cells and have thin muscle fibers, typical markers for immature muscle fiber growth, diminished recruitment, and delayed differentiation (125, 126). Severe disruption of RNA processing is the key element in the diminished capacity of muscle precursor cells in muscle formation in cDM, as recently demonstrated by combining transcriptome profiling of muscle tissue from patients and mouse models (85). In adult-onset DM1, the number of satellite cells are increased in distal but not in proximal muscles (218). Late myogenic differentiation markers are not fully expressed (219). In cell culture, DM1 and DM2 myoblasts show a premature proliferative growth arrest compared with healthy myoblasts (5). Combined, these observations point to a situation in which the regenerative capacity of satellite cells induced in response to fiber dystrophy is constitutively impaired (36, 220).

To understand muscle wasting in greater detail, we first need to know whether the cellular effects of fiber dystrophy are indeed dominant over those of regeneration failure. Then, to deconvolute the complexity of DM further, molecular analyses are needed. First, we need to know whether failure in pools of satellite cells to adequately balance asymmetric and symmetric division and/or subsequent loss of regenerative potency after myogenic commitment could be involved. Underlying mechanisms and differences between DM1 and DM2 muscles therein must be analyzed. Other studies should be concentrated on the loss of functionality, stability, and viability of fibers in DM1 and DM2. Preferably cell- and lineage-tracing studies should be non-invasive and concentrated on the fate of individual myoblasts, myocytes and muscle fibers over longer periods of aging. For obvious reasons, these types of longitudinal analyses of individual cells are virtually impossible for human muscle. However, tracing of cells during development and maintenance in muscles of animal models of DM will also become challenging.

### Premature Muscle Aging in DM

From a clinical perspective, various symptoms of DM1 can be seen as a manifestation of progeria or accelerated aging (221–223), while aging-like symptoms are not as apparent in DM2. The progression of dystrophy in skeletal muscle in DM1 patients shows similarities with sarcopenia, i.e., age-related loss of muscle mass, strength, and function (5). Experimental evidence is mostly indirect and based on descriptive studies, wherein histopathological features such as grouped atrophy, fiber size variability, and central nuclei were investigated in sarcopenic and DM1 muscle (224). Also compelling ultrastructural and molecular evidence was provided, showing that alterations in RNA metabolism in myonuclei from DM1 patients and in aging muscle share similarity (221, 222, 225).

The mechanisms underlying age-related muscle wasting and weakness are probably diverse and not well understood (226). A recent single-fiber proteomics approach showed that the senescence of type 1 and 2 muscle fibers during aging in healthy donors is characterized by several diverging mechanisms. Differential adaptations in cellular carbohydrate and energy metabolism and the networks for protein quality control and proteostasis were among the most conspicuous changes in slow and fast fibers (194). Earlier profiling studies had pointed to a glycolytic to oxidative shift (227) or non-specified overall changes caused by aging in whole human muscles (228). The numerical loss and the loss of functionality of MuSCs, rather than fibers, with aging have attracted until now more attention, as they provide an explanation for the regenerative failure of aged muscle. For more details on the molecular and cellular findings, we refer the reader to comprehensive reviews on this topic (229–231).

Within the networks for muscle regeneration and maintenance during aging, only a few players and processes have been identified that bear direct relevance for DM1 and DM2 pathophysiology. DNA repair is one important issue. Nuclei in resting satellite cells and in muscle fibers are highly efficient in DNA repair through non-homologous end joining, explaining why repeat expansion predominantly occurs in these cells (232). Ongoing somatic expansion of the (CTG•CAG)n and (CCTG•CAGG)n repeats due to DNA repair in quiescent cells may thus be an important factor in impaired muscle regeneration in patients (23). Whether age-induced changes in the production of mitochondrial reactive oxygen species also have an effect must still be analyzed. Accumulation of reactive oxygen species damage is a known contributing factor to repeat expansion (233, 234). Age-dependent changes in oxidative metabolism must, however, have different effects in DM1 and DM2 muscles, as the affected fiber types differ in both forms of disease.

The shortening of telomeres is probably not a major contributor to muscle aging, although effects on premature senescence of DM2 satellite cells have been suggested (220). The situation in DM1 is less clear. Satellite cells in cDM patients did have a higher telomere shortening rate, but they entered senescence before reaching a critical length. This argues against a determining role of telomere shortening as an explanation for diminished differentiation capacity in cDM muscle (218, 235).

A more likely candidate mechanism for the premature growth arrest in DM1 muscle precursor cells is activation of the *p16^Ink4a^*-pathway that leads to CDK4 inhibition and cell cycle arrest. p16 accumulates in myoblasts from DM1 patients in response to (CTG)n-related stress (220, 235), resulting in impaired regeneration and atrophy. As mentioned, aging-like symptoms are not so apparent in DM2 patients and the *p16* pathway appears not to be altered in DM2 satellite cells and fibers (220, 221). Finally, increased p38/MAPK signaling is a typical feature of aged satellite cells (236), but evidence for p38 signaling abnormalities in DM muscle is lacking. Also evidence for the involvement of apoptosis in DM muscle wasting is still limited (159, 163, 237).

An interesting test for the question how DM effects are superimposed on senescence of normal aging would be to study the effects of ablation of *p16^Ink4a^*-expressing cells in muscle of DM mouse models. This is possible with use of a genetic approach recently developed by Baker and co-workers in Van Deursen’s laboratory (238) and also with drug treatment (239). Any alteration in muscle health in the DM mice would provide us with novel insight in the causative effects of expanded repeats on the viability of progenitor cells in muscle.

### Stress Signaling in Adaptation to Regenerative Failure, Effects of Disease, and Aging

Adaptation to cell-autonomous stress in muscle depends on a combination of intrinsic and extrinsic signaling mechanisms. Many intracellular pathways are known that protect cells against stress from for example DNA damage, proteotoxicity, and calcium-mediated excitotoxicity (240). Best-known are the P53, AKT, and NRF2 pathways, but these pathways have not yet been intensely studied in skeletal muscle of DM patients.

Changes in intercellular communication may also fulfill a central role. Many of the secreted hormones and factors that are exchanged between cells and orchestrate myogenesis and regeneration have been extensively discussed in some of the reviews mentioned above (208). Among these are the WNT proteins, HGF, FGFs, IGF-1 splice variants, myostatin, and TGF-β (241). Although the working mode of these secreted factors is reasonably well understood, it is not always clear what cell types in the muscle stem cell niche are in the secretory and/or the responding mode. Satellite cells from cDM patients secrete increased levels of prostaglandin E2 *in vitro*. This secretion is controlled *via* upregulation of cyclo-oxygenase 2, mPGES-1, and prostaglandin E2/EP4 receptors. A direct consequence of the prostaglandin E2 upregulation is a decrease in intracellular Ca^2+^ and impairment of fusogenic capacity of the satellite cells (242). It was also shown that cDM muscle and primary myoblast derived thereof produce a higher level of IL-6, indicative for increased activity of this myokine signaling pathway (52).

Another conspicuous observation was that variation in the level of CELF1, as seen in cDM muscles, causes imbalance in the production of subunits for the signal recognition particle in the ER-secretory pathway (243). CELF1 misregulation may thus be coupled to changes in the secretory route for extracellular matrix proteins. Others confirmed that production of ECM proteins is indeed altered in muscle of a mouse model for DM1 (244). Taken together, this is compelling evidence that the hormonal and ECM environment of progenitor cells in the DM muscle are changed. There is no doubt that this will compromise the “cry-for-help” communication in DM muscle and its adaptive regenerative capacity in response to accelerated fiber decay due to repeat stress.

### MicroRNAs (miRNAs) and Other Non-Coding RNAs in Muscle Homeostasis

MicroRNAs have a critical role in cellular stress responses, differentiation, proliferation, and apoptosis in muscle (245, 246). MiRNAs are short, highly conserved non-coding RNAs that occur in all cell types, where they regulate the stability and the translational efficiency of target mRNAs (247). Multiple miRNAs that regulate differentiation and stress adaptation of skeletal muscle, referred to as myomiRs, exist (248). Among them are miR-1, -133a, -133b, -206 (the most abundant miRNA in skeletal muscle), and miR-208. Expression of these miRNAs is regulated by transcriptional networks involving *MEF2, MYOD, SRF*, and *TWIST1* (249, 250). Non-muscle specific miRNAs that regulate differentiation and regeneration after muscle injury are miR-181, -221, and -222 (251).

Myoblasts and myofibers utilize exosome-clustered extracellular miRNAs as paracrine and endocrine communication signals to regulate homeostasis and regeneration. Extracellular myomiRs are elevated during perinatal muscle development and after exercise-induced muscle regeneration. Also in primary human myoblast and C2C12 cultures, these extracellular myomiRs were elevated and appeared to be released selectively as a consequence of the differentiation process (252).

Myotonic dystrophy type 1 and 2 profiling studies showed that deregulation of intracellular miRNA content in muscle, and extracellular extrusion *via* exosome secretion is a hallmark of disease. Eight miRNAs were found to be significantly deregulated in the serum of DM1 patients (i.e., miR-1, -27b, -133a, -133b, -140-3p, -206, -454, and -574) (253). Earlier work had shown upregulation of miR-1, -206, and -335 and downregulation of miR-29b, -29c, and -33 in DM1 biopsies compared with controls (254, 255). Moreover, cellular distribution of miR-1, -133b, and -206 was altered in DM1 skeletal muscles. Koutsoulidou et al. demonstrated that appearance of miR-1, -133a, -133b, and -206 in serum correlated with the progression of muscle wasting in DM1 patients. All four miRNAs were found encapsulated within exosomes in the circulation (256). Cell and animal model studies suggest that *MBNL* expression is controlled by miR-277 and -304 (257), and miR-30-5p (258), and that this regulatory network could be involved in inhibition of myogenic differentiation in DM1. In DM2 muscle biopsies, the levels of 11 miRNAs were found to be significantly modulated (259). Of these, three also showed modulation in DM1 patients (i.e., miR-193bp, -208a, and -381). Expression levels of the other eight (i.e., miR-34a-5p, -34b-3p, -34c-5p, -125b-5p, -146b-5p, -193a-3p, -221-3p, and -378a-3p) fitted in a unique DM2 profile. The differences in miRNA expression profiles might contribute to the differences in muscle pathobiology between DM1 and DM2 (259).

Long non-coding RNAs and circular RNAs (circRNAs) may also have a role as regulators of muscle homeostasis and gene expression (260, 261). LncRNAs are arbitrarily defined as RNAs >200 nts without overt protein-coding potential, of which at least 5,000 have been identified so far (262). CircRNAs are shaped as covalently closed molecules that lack 5′ and 3′ ends. They are expressed by a high number of genes and are highly conserved among species (263). Although little is known about the function of these RNA species, it has been shown that they can modulate gene expression by competing for miRNA or protein binding, or with regular mRNA production (264–267). Some lncRNAs are important players in muscle differentiation (268, 269) and involved in the pathomechanisms for Duchenne muscular dystrophy (270) and facioscapulohumeral muscular dystrophy (271). *Malat1*, one of the most abundant lncRNAs, was recently found to slow down myogenic differentiation in mice by interference with MyoD-binding loci and formation of a repressive histone-methylation complex. After the onset of differentiation, miR-181 targets *Malat1* RNA for breakdown to release the repression (272). Our group has published evidence that *DM1-AS* transcripts belong to the class of lncRNAs. After alternative splicing and alternative polyadenylation, different (CAG)n repeat containing *DM1-AS* RNA isoforms are produced. Like many other lncRNAs, *DM1-AS* RNA is expressed at very low copy numbers per cell, in parallel with (CUG)n-containing *DMPK* mRNA (273). It remains to be seen whether expanded *DM1-AS* transcripts have an effect on DM1 myopathy, either in isolation or together with expanded *DMPK* transcripts. No circRNAs that are possibly linked to DM have so far been identified, but considering the fast developments, we might soon hear more from this field of research.

## Regenerative Medicine for DM: Progenitor Cells as Source for Muscle Healing

### Use of MuSCs

The satellite cells, the adult MuSCs located between the basal lamina and the sarcolemma of the multinucleated myofibers, form the main pool of progenitors for skeletal muscle regeneration *in vivo* (Table 2). A large body of research has been devoted to the isolation, propagation, and genome tailoring of these cells, as they are the most logical candidates for use in future cell-based therapies, capable of restoring tissue homeostasis, and enhancing muscle repair in patients with myopathies.

#### Identification and Isolation of MuSCs

For an *ex vivo* approach to gene therapy of DM in coupling with muscle cell transplantation the availability of sufficient quantities of MuSCs is a prerequisite. Use of these cells for regenerative medicine in DM, whereby different groups of skeletal muscles are differentially affected, will not be simple. Indeed, although all satellite cells should be considered remnants of embryonic development prepared to recapitulate muscle development in the event of muscle damage (197), it is only a fraction of this heterogeneous population that fully preserves the self-renewal potential and myogenic capacity, when brought in *in vitro* culture. This seemingly stochastic nature of fate adoption, which is associated with a high-degree of heterogeneity and plasticity of the satellite cell population in the natural environment of the muscle (203), is a complicating factor during the period that they regain proliferative activity as myoblasts.

Another complicating factor is that MuSCs have the same embryonic origin as the muscle in which they reside. Most skeletal muscles of the trunk and limb are derived from somites, but head muscles originate from cranial mesoderm. These distinct origins specify distinct genetic programs (274), which may be permanently associated with the intrinsic properties of MuSCs (275). More study is thus needed to verify whether the distinct origin is also a determining and retained factor for capacity to participate in regeneration of muscles in different locations of the body, or whether differences are smoothened out upon maintenance of cells in *in vitro* culture. Lastly, aging of the donor seems to render the MuSC pool increasingly dysfunctional, as MuSCs progressively lose their potency due to cell death and terminal differentiation. Hence, aging forms an extra problem in cases where the patient’s own progenitor cells must be used for cell therapy to circumvent immunological problems, and especially so in patients with late-onset genetic myopathies like in DM2 or certain cases of DM1.

Skuk and colleagues came up with three properties that cells used for repair of damaged and replacement of lost muscle fibers should have: (i) ability to fuse with pre-existing myofibers, (ii) ability to form new myofibers, and (iii) ability to produce myogenically committed stem cells (276). This means that the MuSC’s capacity to participate in all aspects of muscle homeostasis must be maintained during expansion *ex vivo*. Novel strategies for satellite cell culture and preservation of self-renewal capacity before transplantation into muscle have now become available. Cell culture on pliable soft hydrogel matrices, in combination with pharmacological inhibition of p38/MAPK signaling (277) or culture on natural biopolymeric films (278) simulate the conditions of the muscle stem cell niche and help to preserve MuSC quiescence and enhance their self-renewal capacity. Also modulation of *PAX7* expression may thereby be of help (279).

#### Transplantation of MuSCs: Preclinical Studies Only

**Figure 4:**
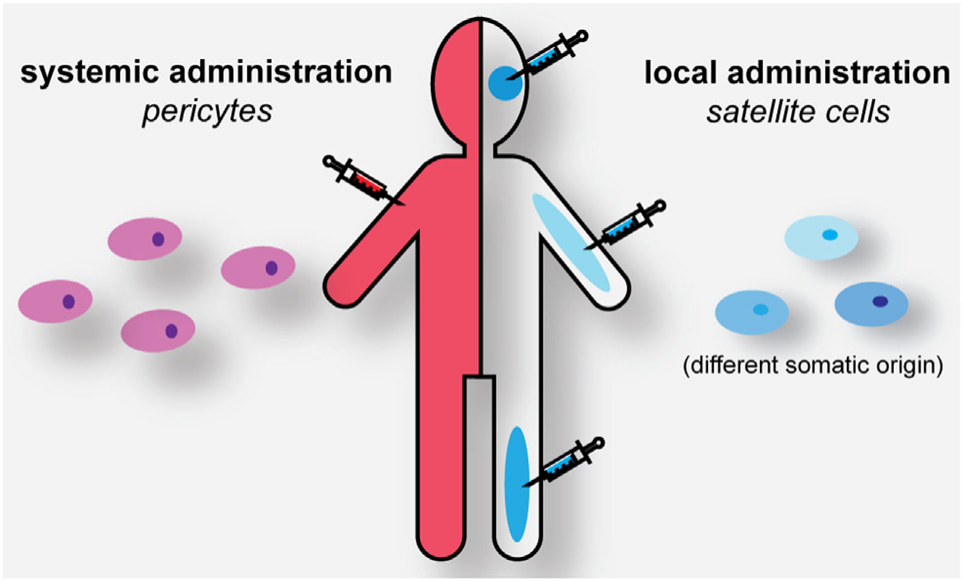
Strategies for cell-based muscle therapy in myotonic dystrophy. Genome-edited autologous or HLA-matched pericytes (PCs) can be administered systemically for muscle healing. Genome-edited or HLA-matched satellite cells need to be engrafted locally in the corresponding muscles to have a regenerative effect.

Currently, the use of MuSCs in cell-based therapies is almost impossible. As demonstrated in animal model studies, MuSCs cannot be delivered systematically to all muscles in the body (280). Upon intravenous delivery they accumulate in the lung, liver, spleen, and kidney but not in skeletal muscle. One of the largest technical hurdles that limit the feasibility of MuSC transplantation is, therefore, associated with the route of administration, i.e., intramuscular injection (Figure 4). Initial trials aiming to regenerate skeletal muscle by local injection of donor myoblasts failed due to their poor survival and limited ability to migrate more than a few millimeters away from the site of injection (281–283). Upon engraftment, these satellite-derived myoblasts could not efficiently repopulate the satellite cell niche, and therefore were not able to contribute significantly to muscle regeneration (284, 285). Future work is necessary to find out whether some of these issues might be overcome by increasing the numbers of engrafted cells, or by better preservation of their stemness during *in vitro* propagation as discussed above.

Further work on MuSCs in culture is, therefore, necessary. For application in basal and translational research in DM, immortalized myoblast cell lines are available. These lines have preserved the molecular hallmarks of disease, including splicing abnormalities and repeat RNA-MBNL foci (86) and were generated by lentiviral-mediated expression of the catalytic subunit of the human telomerase (*TERT*) and *CDK4*, the natural p16 ligand. Immortalized cells constitute an unlimited source of cells for evaluation of compounds with therapeutic potential. Immortalization *per se* may not be detrimental for the ability of muscle progenitor cells to serve in therapeutic engraftment experiments in mice, as already shown earlier for these type of cells and for SV40-TAg^ts^ immortalized cells (286, 287). However, for obvious reasons use of these transformed cells for human studies will probably remain restricted for *in vitro* work.

### Use of Stem Cells From Non-Muscle Origin

The continuous search for stem cells with potency for transdifferentiation and adaptation from other sites than within the muscle basal lamina (288–295) has led to the identification of entirely unexpected cell types with muscle progenitor capacity. Among these are the vessel-associated MABs and PCs the best-known examples (Figure 3) (199).

#### Identification and Isolation of PCs and MABs

The participation of MABs and PCs in myogenic differentiation and regeneration *in vivo* is still a poorly understood phenomenon. There is, however, compelling evidence that these cell types have great potential for boosting muscle repair in regenerative medicine. One advantage, which MABs and PCs may have, is that they rapidly acquire unlimited lifespan and maintenance of multipotency, making them ideally suitable for the generation of replenishable pools of transplantable cells. Skeletal muscle tissue itself is the most effective source for PCs with this potential (200, 296). Their isolation can be accomplished by using explant culture methodology (297, 298), eventually in combination with enzymatic dissociation and FACS for surface markers (200, 299). PCs with skeletal myogenic potential can be distinguished by expression of *ALP* (200, 300) and new biomarkers for therapeutic potency, like *PW1/Peg3*, a regulator of myogenic ability and migration capacity in PCs, MABs and satellite cells, have recently been identified (301). Expression of *PW1/Peg3* is high in both MABs and PCs and its level of expression correlates with their progenitor cell competence. Moreover, lack of *PW1/Peg3* expression abrogates the cells ability to cross the vessel wall and to engraft into damaged myofibers through the modulation of the junctional adhesion molecule. PCs and MABs are expandable *in vitro* as a relatively homogeneous population and transducible with viral vectors for genomic editing.

#### Engraftment of PCs and MABs

Pericytes and MABs are able to systemically reach the target tissue, where they engraft and differentiate toward the myogenic lineage (Figure 4). One possible complication, however, is that adequate measures are necessary to ensure that myogenic commitment of these vessel-derived progenitor cells is appropriately stimulated, while adipose and fibrogenic commitment must be avoided. Several recent publications have implicated a role for a PC subtype in fibro-adipose infiltration of tissues (299, 302). Consistent with age-dependent changes in regeneration capacity seen before, this property seems to be more present in PCs isolated from aged individuals. PCs failed to differentiate or participate in myofiber repair following injury, but contributed to enhanced fibrous tissue deposition within the interstitial space in aged muscle (299, 303–305). Further work is thus necessary to see whether PCs and MABs are truly the ideal candidates for use in regenerative medicine in DM patients.

Translational studies in the GRMD dog model of myopathy demonstrated that *ex vivo* cultivated PCs can indeed adopt myogenic fate when exposed to injury factors *in vivo* and are able to directly differentiate into skeletal muscle or replenish the SC pool *via* activation of *Pax7, Myf5*, or *MyoD* at the onset of differentiation (200). For the GRMD model “a remarkable clinical amelioration and preservation of active motility” was seen (306). The first human clinical study with PCs was published in 2015, investigating primarily the safety of intra-arterial transplantation of *HLA*-matched donor cells. This exploratory clinical trial was performed in five Duchenne patients, in combination with immunosuppressive therapy. Clinical laboratory and MRI analysis revealed that the study was relatively safe. Unfortunately, the effects of the cellular therapy on muscle function were inconclusive.

Although the possibility for systemic administration is one of the strongest arguments for preference of vessel-associated progenitor cells over satellite cells, there is also concern, as blood flow in the artery of microvasculature downstream of the injection site might get disrupted (307). Moreover, a fraction of the injected cells might become trapped in filter organs decreasing the amount of cells available for engraftment into dystrophic muscle (200). Modification to improve homing to damaged muscles (308) or altering cell surface (309) needs to be studied in more detail to address these possible problems. For DM, research regarding the potential and use of MABs or PCs for therapy is entirely missing.

#### Induced Pluripotent Stem Cells (iPSCs)

Also whole new approaches toward deriving myogenic progenitor cells from pluripotent embryonic stem cells and iPSCs are now being developed (310–315). Generation of iPSCs from fibroblasts of DM1 and DM2 patients has been published (316–321). Recently, a revolutionary new method to direct human iPSCs to adopt muscle progenitor cell identity and create a renewable source of muscle progenitors for regenerative medicine was developed. Hicks et al. found that the use of FACS of cells for two cell surface markers, *ERBB3* and *NGFR*, and treatment with a TGFβ inhibitor gave an enormous enrichment for progenitors with regenerative potential during engraftment (322). Further work is necessary to verify whether simple extrapolation of these animal model transplantation findings to the human situation is possible.

### Cell-Based Therapy in Combination With Genome Editing

To prevent immunological problems linked to MuSC transplantation, the use of progenitor cells from HLA-matched donors or autologous cells from patients is strongly advisable. For DM1 and DM2 cells, this implicates that genome editing must be employed to normalize the length of the expanded repeats or the synthesis of the toxic RNAs must be otherwise permanently prevented. With the advent of gene editing tools such as ZFN, TALEN, and CRISPR/Cas9 this now has become a realistic goal. Specifically for DM1, a small number of gene editing studies have been published recently, all aiming at the prevention of the presence of toxic, expanded repeat-containing RNA.

Gao et al. inserted a poly(A) signal upstream of the expanded (CTG)n repeat in *DMPK* in iPSCs. This insertion led to premature termination of transcription and prevented production of (CUG)n repeat containing transcripts. As the *DMPK* mRNAs were now missing the repeat-containing 3′ end, a healthy stem-cell pool was created (320). Cardiomyocytes derived from these iPSCs reverted to normal splicing for a number of pre-mRNAs known to be misspliced in DM1.

Pinto et al. used a deactivated Cas9 variant to impede synthesis of expanded (CUG)n RNA during transcription (323), while Batra et al. (324) used an RNA-targeting Cas9 to eliminate toxic expanded RNA after production. Both studies showed efficient elimination of cellular hallmarks of disease, but the strategies used seem not well suited for permanent transformation of muscle progenitor cells and prevention of repeat RNA effects.

More permanent effects for use in cellular strategies may be expected from removal or trimming of the (CTG•CAG)n repeat expansion in the DM1 locus, creating permanently normalized *DMPK*/*DM1-AS* alleles. Our own group and others have published that excision of the repeat (and short flanking sequences) can be achieved by dual CRISPR/Cas9 cleavage at either side of the repeat (113, 325). Repeat removal had no adverse biological effects on DMPK isoform production and normalized splicing and myogenic capacity. Notably, CRISPR/Cas9 cleavage in the vicinity of the repeat was associated with a risk of uncontrollable DNA rearrangements across the area (113, 325). Also off-target alteration elsewhere in the genome is a known danger in the application of CRISPR/Cas9 technology. Hence, careful characterization and selection of cell clones with only the desired genome alterations should become routine steps in future cell-based therapeutic strategies.

Use of repeat-corrected cell therapy may serve to halt the degenerative process, or delay or prevent the onset of disease when applied upon first diagnosis with DM. In parallel, more work will be devoted to the development of modalities for direct *in vivo* treatment of DM, with vector-mediated gene-editing therapy. Finding ways for improvement of the quality of life of patients with DM will remain the goal of a large variety of future translational studies.

## Author Contributions

LA and CA designed the figures. LA, CA, and BW drafted the contents of this review, and together with DW wrote the text. All authors contributed equally to critical reading of the final manuscript, including text and figures.

## Conflict of Interest Statement

The authors declare that the research was conducted in the absence of any commercial or financial relationships that could be construed as a potential conflict of interest.
